# Metabolomics Analysis of Sodium Salicylate Improving the Preservation Quality of Ram Sperm

**DOI:** 10.3390/molecules29010188

**Published:** 2023-12-28

**Authors:** Haiyu Bai, Zhiyu Zhang, Wenzheng Shen, Yu Fu, Zhikun Cao, Zibo Liu, Chao Yang, Shixin Sun, Lei Wang, Yinghui Ling, Zijun Zhang, Hongguo Cao

**Affiliations:** 1 College of Animal Science and Technology, Anhui Agricultural University, Hefei 230036, China; 2 Anhui Province Key Laboratory of Local Livestock and Poultry Genetic Resource Conservation and Bio-Breeding, Anhui Agricultural University, Hefei 230036, China

**Keywords:** sheep, sperm, sodium salicylate, low-temperature preservation, ultrastructure, metabolomics

## Abstract

The aim of this study was to investigate the effects of sodium salicylate (SS) on the preservation and metabolic regulation of sheep sperm. Under 4 °C low-temperature conditions, SS (at 10 µM, 20 µM, 30 µM, and 50 µM) was added to the semen diluent to detect sperm motility, plasma membrane, and acrosome integrity. Based on the selected optimal concentration of SS (20 µM), the effects of 20 µM of SS on sperms’ antioxidant capacity and mitochondrial membrane potential (MMP) were evaluated, and metabolomics analysis was conducted. The results showed that on the 20th day of low-temperature storage, the sperm motility of the 20 µM SS group was 62.80%, and the activities of catalase (CAT) and superoxide dismutase (SOD) were significantly higher than those of the control group (*p* < 0.01). The content of Ca^2+^, reactive oxygen species (ROS), and malondialdehyde (MDA) were significantly lower than those of the control group (*p* < 0.01), and the total antioxidant capacity (T-AOC) was significantly higher than that of the control group (*p* < 0.05); mitochondrial activity and the total cholesterol (TC) content were significantly higher than those in the control group (*p* < 0.01). An ultrastructural examination showed that in the SS group, the sperm plasma membrane and acrosome were intact, the fibrous sheath and axoneme morphology of the outer dense fibers were normal, and the mitochondria were arranged neatly. In the control group, there was significant swelling of the sperm plasma membrane, rupture of the acrosome, and vacuolization of mitochondria. Using metabolomics analysis, 20 of the most significant differential metabolic markers were screened, mainly involving 6 metabolic pathways, with the amino acid biosynthesis pathway being the most abundant. In summary, 20 µM of SS significantly improved the preservation quality of sheep sperm under low-temperature conditions of 4 °C.

## 1. Introduction

With the widespread application of artificial insemination technology, the sperm of excellent male breeding animals, as an important genetic resource, has broken through time and geographical limitations, greatly improving reproductive efficiency [1]. The effective preservation of semen and the quality of sperm preservation are key factors determining the success of artificial insemination. The principle of sperm preservation is to inhibit the metabolism of sperm at low temperatures, achieving an extension of sperm survival times. In breeding practice, low-temperature preservation under 4 °C conditions is simple, convenient, and fast, making it easy to promote and apply. For ram sperm, due to the presence of a large amount of polyunsaturated fatty acids (PUFAs) in the sperm plasma membrane, they are highly sensitive to low temperatures [2,3,4] and susceptible to the influence of reactive oxygen species (ROS), which, in turn, leads to lipid peroxidation [5]. Under physiological conditions, ROS plays an important role in maintaining the physiological function of sperm [6,7], such as sperm capacitation, acrosome reactions, and mitochondrial membrane stability [8]. However, excessive ROS can lead to oxidative stress in sperm, disrupting the fluidity of the sperm plasma membrane and the integrity of nuclear DNA [9]. At present, it is widely believed that oxidative stress caused by ROS is the main reason for the quality decline in sperm during preservation [10,11]. Therefore, adding antioxidants to semen diluents to eliminate the damage of free radicals to sperm has become a focus of current research.

Sodium salicylate (SS) is a derivative of aspirin, which has been used as a non-steroidal anti-inflammatory drug for hundreds of years due to its analgesic and anti-inflammatory effects [12]. Studies have shown that SS can protect rats from oxidative stress induced by rotenone and increase the activity of superoxide dismutase (SOD) [13]. Meanwhile, SS can eliminate free radicals produced by the liver and reduce the production of malondialdehyde (MDA) [14]. In addition, salicylates have been reported as activators of AMP-activated protein kinase (AMPK), significantly increasing AMPK activity by increasing phosphorylation at the Thr172 site [15]. AMPK is a key molecule in regulating energy metabolism and is crucial for maintaining the balance of cellular physiological activities [16]. In sperm metabolism, AMPK inhibits the ATP consumption pathway, accelerates the ATP synthesis pathway, maintains both the mitochondrial membrane potential (MMP) and plasma membrane integrity of sperm, and regulates sperm motility [17,18]. In this study, based on the multiple biological functions of SS, sheep sperm was taken as the research object to explore the protective effect of the AMPK activator SS on sheep sperm at low temperatures, and its related metabolic regulation mechanisms were analyzed. In addition, the AMPK inhibitor compound C (CC) [19,20] was used for validation, providing a theoretical basis for the metabolic regulation of SS in semen preservation.

## 2. Results

### 2.1. Effects of SS on Sperm Quality

Motility, plasma membrane, and acrosome integrity are important indicators of sperm quality. Adding 10 μM, 20 μM, 30 μM, and 50 μM SS to the diluent had varying degrees of protective effects on sperm motility, plasma membrane, and acrosome integrity. The effect of SS on sperm quality is shown in Table 1. On the 2nd to 4th day of sperm preservation, the sperm motility of the 20 μM and 30 μM SS groups was significantly higher than that of the SS control group (*p* < 0.05). On the 10th to 18th day, the sperm motility of the 10 μM, 20 μM, and 30 μM SS groups was significantly higher than that of the SS control group (*p* < 0.05). On the 20th day, the sperm motility of the 10 μM, 20 μM, 30 μM, and 50 μM SS groups was significantly higher than that of the SS control group (*p* < 0.05). Among them, the sperm motility of the 20 μM SS group was 62.80 ± 2.29%, which is significantly higher than that of the other groups (*p* < 0.05).

**Figure 1:**
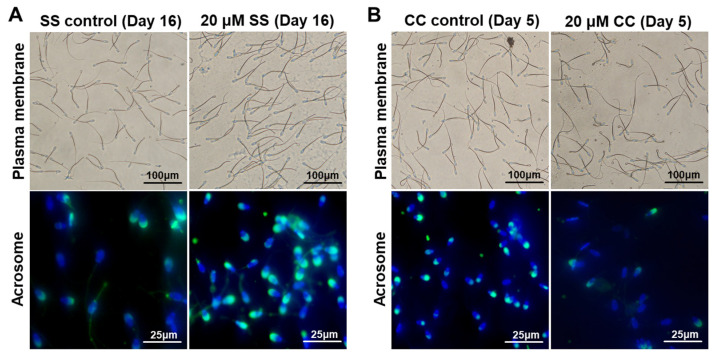
Effects of sodium salicylate (SS) and compound C (CC) on sperm plasma membrane integrity and acrosome integrity after storage at 4 °C. (**A**) The effect of 20 μM of SS (Day 16). (**B**) The effect of 20 μM of CC (Day 5). The curvature of the sperm tail indicates that the sperm plasma membrane is intact. Green fluorescence represents sperm acrosome staining, while blue fluorescence represents sperm DNA staining.

On the 2nd to 6th day of sperm preservation, the sperm plasma membrane integrity rates of the 20 μM and 30 μM SS groups were significantly higher than those of the SS control group (*p* < 0.05). On the 10th to 20th day, the sperm plasma membrane integrity rates of the 10 μM, 20 μM, and 30 μM SS groups were significantly higher than those of the SS control group (*p* < 0.05), with the 20 μM SS group having the highest sperm plasma membrane integrity rate (Figure 1A).

After being stored for 4 days, the sperm acrosome integrity rate showed a certain pattern. On days 6–8, the sperm acrosome integrity rate in the 20 μM, 30 μM, and 50 μM SS groups was significantly higher than that in the SS control group (*p* < 0.05). On days 10–20, the sperm acrosome integrity rate in the 20 μM and 30 μM SS groups was significantly higher than that in the SS control group (*p* < 0.05), with the 20 μM SS group having the highest sperm acrosome integrity rate. In summary, SS has a protective effect on sperm, and higher concentrations of SS do not provide additional protection for sperm. The best protective effect is achieved with 20 μM SS.

### 2.2. Effects of CC on Sperm Quality

The impact of CC on sperm quality is shown in Table 2. On the 3rd to 4th day of preservation, the sperm motility of the 20 μM CC group was significantly lower than that of the CC control group (*p* < 0.05), while there was no significant difference between the 1 μM, 5 μM, and 10 μM CC groups and the CC control group (*p* > 0.05). After 4 days of preservation, sperm motility decreased with the increase in the CC concentration. On the 6th to 7th day, the sperm motility of the 20 μM CC group was significantly lower than that of the 1 μM, 5 μM CC, and CC control groups (*p* < 0.05).

After 4 days of preservation, the sperm plasma membrane integrity rate showed a certain pattern. On days 5–7, the sperm plasma membrane integrity rates in the 5 μM, 10 μM, and 20 μM CC groups were significantly lower than those in the CC control group (*p* < 0.05), while there was no significant difference between the 1 μM CC group and the CC control group (*p* > 0.05). On day 7, the sperm plasma membrane integrity rate in the 20 μM CC group was significantly lower than that in the other groups (*p* < 0.05) (Figure 1B).

In total, 20 μM of CC first showed damage to the sperm acrosome, and on the 3rd to 4th day of preservation, the sperm acrosome integrity rate in the 20 μM CC group was significantly lower than that in the other groups (*p* < 0.05). On the 5th to 7th day, the acrosome integrity rates of the 1 μM, 5 μM, 10 μM, and 20 μM CC groups were significantly lower than those of the CC control group (*p* < 0.05), while the 20 μM CC group had the lowest acrosome integrity rate.

In summary, as the concentration of CC increased, the quality of the sperm further decreased, and 20 μM of CC is the optimal concentration to verify the protective effect of the AMPK activator SS on sperm.

### 2.3. Effects of SS and CC on the Antioxidant Ability

**Figure 2:**
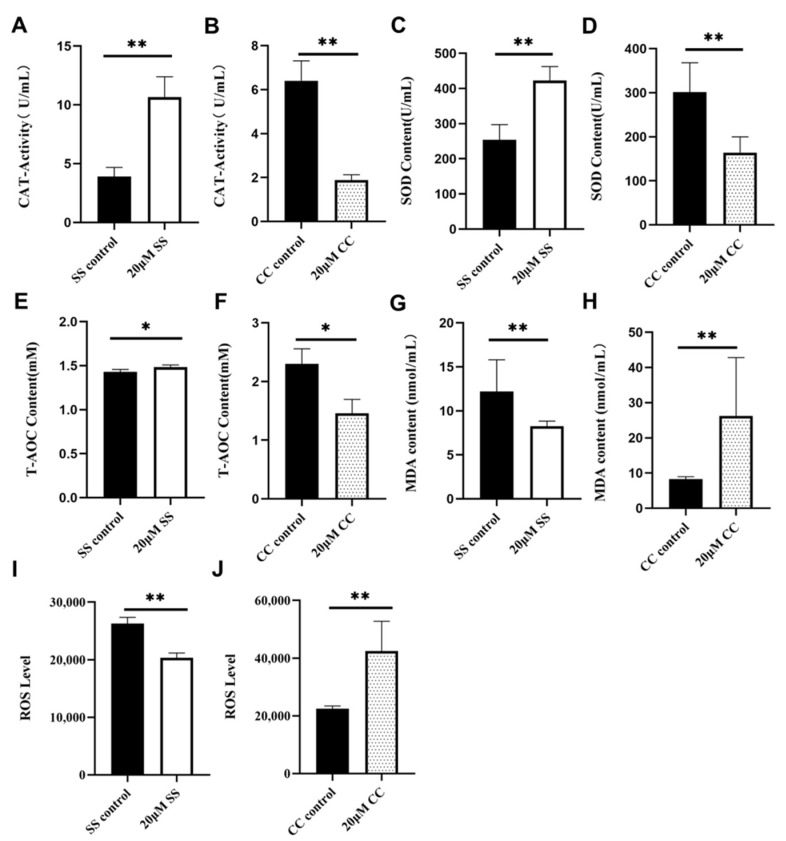
Detection of antioxidant ability of sperm. (**A**) CAT content in 20 μM SS group. (**B**) CAT content in CC group. (**C**) SOD content in 20 μM SS group. (**D**) SOD content in CC group. (**E**) T-AOC level in SS group. (**F**) T-AOC level in CC group. (**G**) MDA level in SS group. (**H**) MDA level in CC group. (**I**) ROS level in the SS group. (**J**) ROS level in CC group. * *p* < 0.05, ** *p* < 0.01. CAT, catalase; SOD, superoxide dismutase; T-AOC, total antioxidant capacity; MDA, malonaldehyde; ROS, reactive oxygen species.

Through the detection of sperm motility, plasma membrane, and acrosome, it was found that the optimal concentrations of SS and CC were both 20 μM. Subsequently, the antioxidant capacity of sperm in the 20 μM SS group was tested (the SS group was tested on the 16th day; the CC group was tested on the 5th day). In total, 20 μM of CC was used to confirm the impact of SS on sperms’ antioxidant capacity. The final results are shown in Figure 2. Catalase (CAT) and SOD are important antioxidant enzymes in the sperm antioxidant system; 20 μM of SS significantly increased the activity of CAT and SOD in sheep sperm (*p* < 0.01, Figure 2A,C), while 20 μM of CC significantly reduced the activity of CAT and SOD in sperm (*p* < 0.01, Figure 2B,D). Total antioxidant capacity (T-AOC) is an important indicator for testing the total antioxidant function of sperm. In total, 20 μM of SS significantly increased the T-AOC level of sperm (*p* < 0.05, Figure 2E), and 20 μM of CC significantly reduced the T-AOC level (*p* < 0.05, Figure 2F). MDA is one of the products of cell membrane lipid peroxidation, and its production can also exacerbate membrane damage, directly reflecting the degree of membrane damage. The degree of membrane system damage is determined by detecting the MDA content in semen. ROS is a product of cellular metabolism that can induce the peroxidation reaction of PUFAs and damage the sperm plasma membrane. The results show that 20 μM of SS significantly reduced the sperm MDA content (*p* < 0.01, Figure 2G), while 20 μM of CC significantly increased the MDA content (*p* < 0.01, Figure 2H). In total, 20 μM of SS significantly reduced sperm ROS levels (*p* < 0.01, Figure 2I), while 20 μM of CC significantly increased the ROS content (*p* < 0.01, Figure 2J).

### 2.4. Effects of SS and CC on Mitochondrial Energy Metabolism

**Figure 3:**
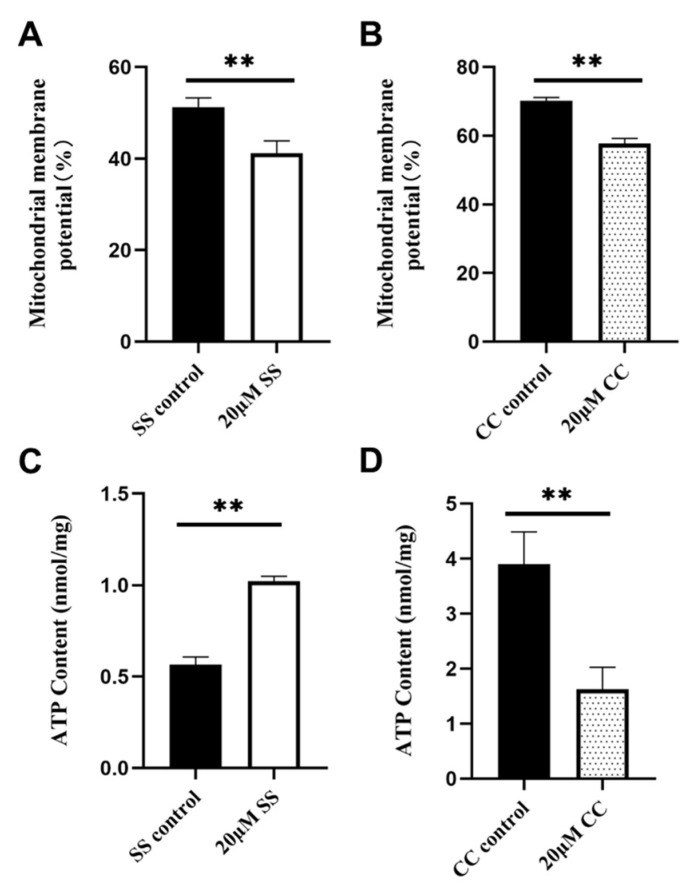
Detection of sperm MMP and ATP levels. (**A**) MMP level in SS group. (**B**) MMP level in CC group. (**C**) ATP content in SS group. (**D**) ATP content in CC group. ** *p* < 0.01. MMP, mitochondrial membrane potential.

**Figure 4:**
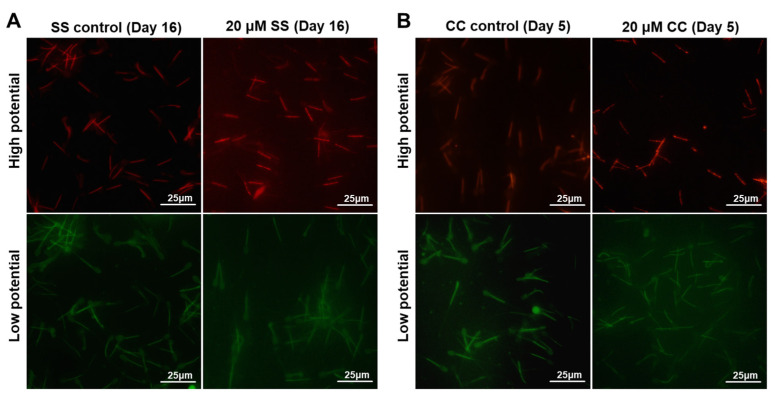
Fluorescence of MMP with JC-1 probe. (**A**) The effect of SS on MMP of sperm (Day 16). (**B**) The effect of CC on MMP of sperm (Day 5). Red fluorescence indicates high membrane potential staining, and green fluorescence indicates low membrane potential staining.

MMP is an important indicator of mitochondrial functional integrity. The high potential of sperm in the 20 μM SS group was evaluated and analyzed. The results showed that the MMP in the 20 μM SS group was significantly higher than that in the SS control group (*p* < 0.01) (Figure 3A), and the MMP in the 20 μM CC group was significantly lower than that in the CC control group (*p* < 0.01) (Figure 3B). In Figure 4, red fluorescence represents the polymer formed by JC-1, indicating a higher MMP, while green fluorescence represents that the monomer JC-1 was present, indicating a lower MMP. ATP is the energy source of sperm. In addition, 20 μM of SS effectively increased sperm ATP levels (*p* < 0.01, Figure 3C), while 20 μM of CC significantly reduced ATP levels (*p* < 0.01, Figure 3D).

### 2.5. Ca^2+^ and TC Content Detection

**Figure 5:**
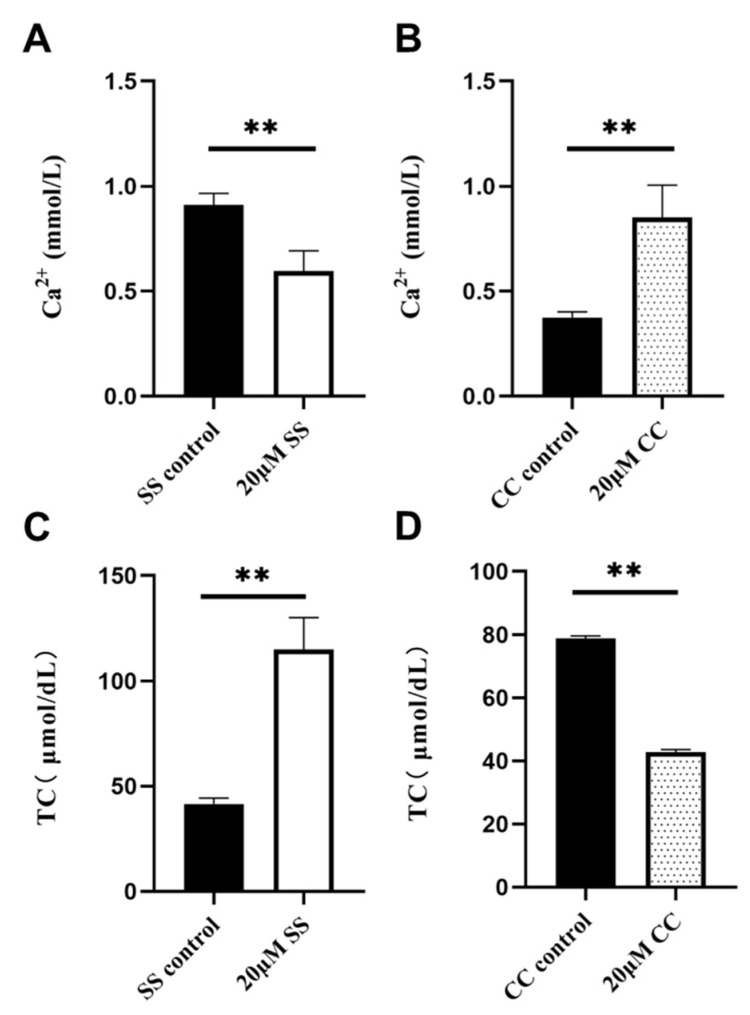
The effect of SS on the content of Ca^2+^ and TC in sheep sperm. (**A**) The Ca^2+^ level in the SS group. (**B**) The Ca^2+^ level in the CC group. (**C**) The TC content in the SS group. (**D**) The TC content in the CC group. ** *p* < 0.01. TC, total cholesterol.

Ca^2+^ is closely related to sperm motility, capacitation, and acrosome reactions. As shown in Figure 5, 20 μM of SS reduced the sperm Ca^2+^ level (*p* < 0.01, Figure 5A), while 20 μM of CC increased the sperm Ca^2+^ level (*p* < 0.01, Figure 5B). Cholesterol regulates plasma membrane fluidity and has a protective effect on the plasma membrane at low temperatures. In total, 20 μM of SS effectively increased the sperm total cholesterol (TC) content (*p* < 0.01, Figure 5C), and 20 μM of CC significantly reduced the TC content (*p* < 0.01, Figure 5D).

### 2.6. Ultrastructural Analysis

**Figure 6:**
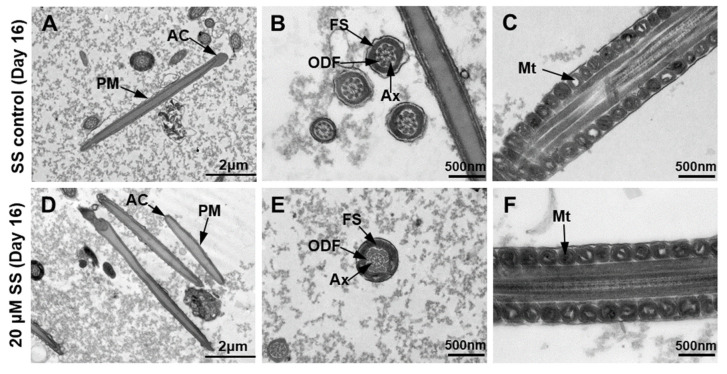
Ultrastructure of sheep sperm under transmission electron microscopy. (**A**) Longitudinal section of SS control group sperm. (**B**) Cross section of sperm tail in the control group. (**C**) Sperm mitochondria in the SS control group. (**D**) Longitudinal section of 20 μM SS sperm group. (**E**) Cross section of sperm tail in the 20 μM SS group. (**F**) Sperm mitochondria in the 20 μM SS group. AC, acrosomal cap; PM, plasma membrane; FS, fibrous sheath; ODF, outer dense fiber; Ax, axoneme; Mt, mitochondrion.

A sperm transmission electron microscopy examination showed that in the SS control group, the plasma membrane of the sperm was swollen, and the acrosome cap was deformed (Figure 6A). In the transverse section of the tail, the fibrous sheath and axoneme morphology of the outer dense fibers were normal (Figure 6B), but the structure of the mitochondria was blurry and showed vacuolization. The mitochondrial cristae disappeared, and the rupture of the middle plasma membrane of the sperm was more distinguished (Figure 6C). Compared with the SS control group, in the 20 μM SS group, the sperm plasma membrane and acrosome were relatively intact (Figure 6D), the fibrous sheath and axoneme morphology of the outer dense fibers were normal (Figure 6E), the mitochondria were arranged neatly, and the mitochondrial cristae were clearly visible (Figure 6F).

### 2.7. Metabolomics Analysis

**Figure 7:**
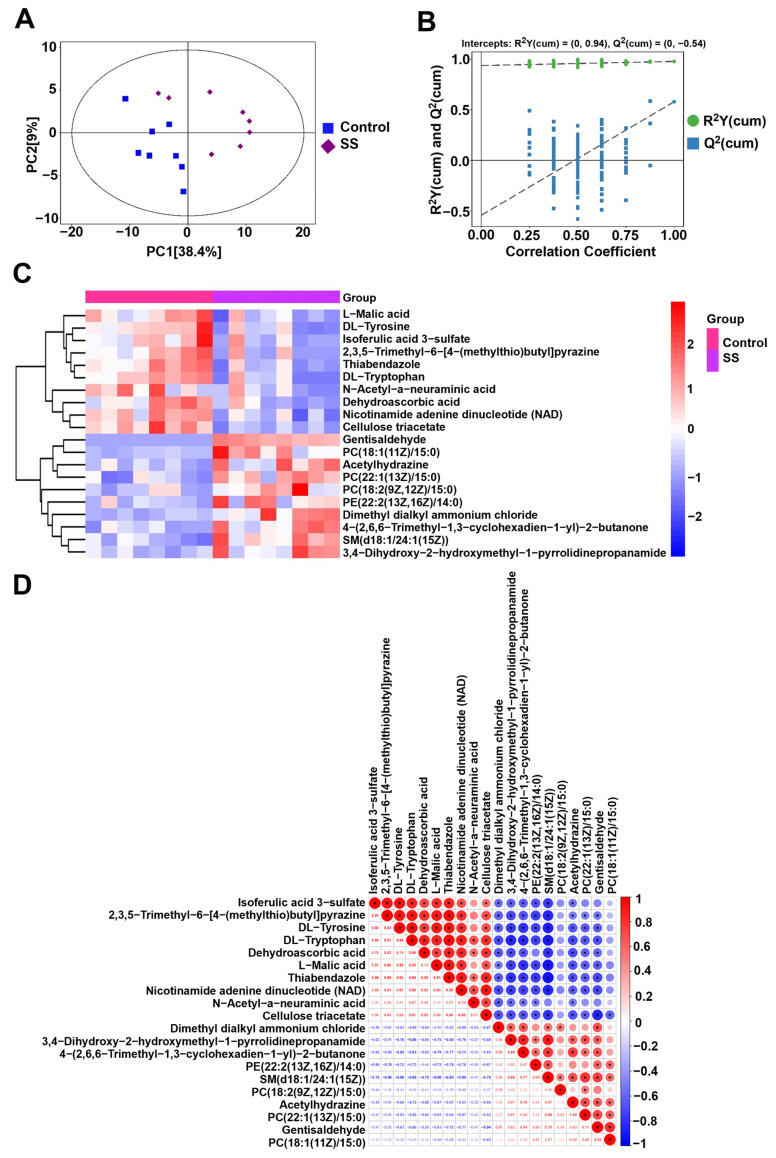

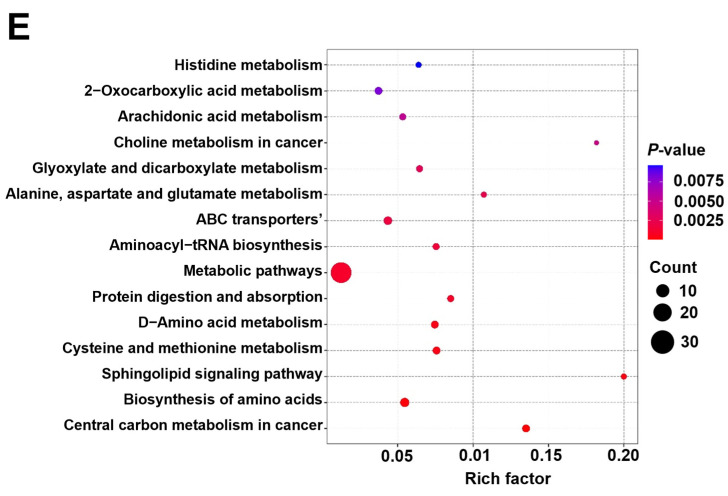
Sperm metabolomic profiling. (**A**) PCA score plot of sperm. (**B**) Score plot of permutation test for OPLS-DA. (**C**) Hierarchical cluster analysis thermogram of metabolites in different groups. (**D**) Correlation analysis of differential metabolites in sperm. * indicates significant correlation. (**E**) KEGG enrichment analysis of sperm metabolites in the 20 μM SS group.

To understand the overall distribution trend of sperm metabolites between the SS control group and the SS group, principal component analysis (PCA) was used to determine the degree of separation between the experimental group and the SS control group. Figure 7A shows that the separation effect of metabolites between the SS control group and the SS group was good. In addition, the displacement test of the OPLS-DA model showed R^2^ > Q^2^, indicating that the metabolomic data had good stability and reproducibility (Figure 7B).

Based on *p* < 0.05 and VIP > 1, differential metabolites were screened. Among them, 138 metabolites induced by SS showed significant changes in the SS control group and 20 μM SS sperm group (Supplementary Table S1 and Figure S1). Hierarchical clustering analysis was performed on the differential metabolites of sperm, as shown in Figure 7C. Red indicates the upregulation of the metabolite expression, and blue indicates the downregulation of the metabolite expression. Differential metabolites such as L-Malic acid, DL-Tyrosine, and Isoferulic acid 3-sulfonate were downregulated in the SS group, while Gentisaldehyde, PC (18:1 (11Z)/15:0), and Acetylhydrazine were upregulated.

The closer the absolute value of the correlation r between differential metabolites to 1, the stronger the correlation is between differential metabolites. There was a positive correlation between DL-Tyrosine and 2,3,5-Trimethyl-6-[4-(methylthio) butyl] pyridine (r = 0.92), Isoferulic acid 3-sulfonate and DL-Tyrosine (r = 0.93), and Thiabendazole and nicotinamide adenine dinucleotide (NAD) (r = 0.90). On the contrary, there was a negative correlation between SM (d18:1/24:1 (15Z)) and L-Malic acid (r = −0.90), SM (d18:1/24:1 (15Z)) and Thiabendazole (r = −0.93), and Gentisaldehyde and Cellulose triacetate (r = −0.84) (Figure 7D).

KEGG analysis was performed on differential metabolites, and 89 metabolic pathways were identified (Supplementary Table S2), mainly including metabolic pathways, ABC transporters, the biosynthesis of amino acids, and other pathways (Figure 7E). Topological analysis was conducted on differential metabolites, and 25 metabolic pathways with the highest correlation were further screened (Supplementary Table S3). Taking into account both enrichment analysis and topology analysis, six main metabolic pathways were identified for analysis, and six different metabolites, L-glutamine, L-histidine, L-aspartic acid, glycolic acid, citric acid, and L-phenylalanine, were detected (Table 3).

## 3. Discussion

SS is a metabolite of aspirin and is widely used in related diseases such as analgesia and as an anti-inflammatory [21,22]. SS also has the ability to inhibit the cyclooxygenase and NF-κB pathway and other functions [23,24]. Studies have found that SS can promote the phosphorylation of the threonine 172 site and directly activate AMPK [25]. In addition, SS, as a chemical capture agent for hydroxyl radicals, has the function of clearing ROS [14,26]. This study found that adding SS can significantly improve the antioxidant capacity of sheep sperm, thereby extending the preservation time and quality of semen.

Mitochondria are the main source of sperm energy and the main site of ROS production [27]. ROS can damage sperm mitochondria, alter MMP, induce mitochondrial dysfunction, and further affect sperm energy metabolism [28]. During sperm preservation, the AMPK signaling pathway can significantly improve sperm preservation quality and antioxidant capacity [29]. After AMPK is activated, it regulates a series of energy metabolism pathways, such as glycolysis, lipid homeostasis, and mitochondrial homeostasis, by phosphorylating key proteins [16]. Hawley et al. demonstrated that SS and its derivatives regulate mitochondrial metabolic processes by activating the AMPK signaling pathway, thereby maintaining an intracellular energy balance [25]. Our study also confirmed that the addition of the AMPK activator SS can significantly improve the mitochondrial activity and antioxidant capacity of sperm.

Due to the high content of unsaturated fatty acids in the sperm plasma membrane, sperm are highly sensitive to oxidative stress. Excessive peroxidation can damage the sperm plasma membrane, leading to a decrease in sperm quality [30]. Cholesterol is the main component of the sperm plasma membrane, and a higher content of cholesterol in the plasma membrane makes it more resistant to cold shock [31,32,33]. Studies have shown that AMPK is involved in the maintenance of sperm plasma membrane, which can inhibit the process of inducing the membrane lipid disorder and reduce the translocation of phosphatidylserine to the outer surface of sperm [34]. We found in the experiment that the cholesterol content in the sperm plasma membrane increased with the addition of the AMPK activator SS, and transmission electron microscopy showed that the plasma membrane was intact and well encapsulated.

The axoneme of mammalian sperm is composed of 9 + 2 arranged microtubule pairs and hundreds of helper proteins [35]. It is known that the motility of mammalian sperm is mediated by motor proteins, which act as motor proteins attached to the double helix microtubules in a circular arrangement in the flagella [36]. In the middle segment of mammalian sperm, mitochondria rearrange into tubular structures, with spirals distributed in the front of the axoneme [37,38]. We found through experiments that under transmission electron microscopy, the sperm axoneme and microtubule system with the addition of SS were normal, and the mitochondrial structure was clear. In the control group, the plasma membrane of the middle segment of the sperm swelled and ruptured, and the mitochondrial structure was blurry. From this, it can be seen that SS can improve sperm quality by protecting the motility system of sperm.

Through metabolomics analysis, it was found that the most significant difference between the SS control group and the SS group mainly involved the biosynthesis of the amino acid metabolic pathway. The differential metabolites involved mainly included glutamine, histidine, phenylalanine, and tryptophan, which mainly involve energy metabolism pathways such as glycolysis, citric acid cycle, and pentose phosphate metabolism, providing energy support for sperm movement. Sperm is affected by oxidative stress during in vitro preservation, and glutamine can promote the sperm synthesis of glutathione, effectively protecting sperm from oxidative stress-induced damage and improving their antioxidant capacity [39,40,41]. In this study, the differential metabolites upregulated by sperm were mainly concentrated in the lipid fraction, such as phosphatidylcholine (PC) and sphingomyelin (SM). We speculated that the sphingolipid signaling pathway may be an important pathway affecting sperm preservation efficiency. NAD is a key factor involved in various forms of cellular metabolism and plays an important role in energy metabolism, DNA repair, and antioxidant activity [42,43]. NAD is consumed and degraded by some enzymes during the metabolic process, and maintaining NAD levels is crucial for mitochondrial homeostasis [44]. Our study found that NAD was significantly downregulated in the identification of differential metabolites in sperm, suggesting that its downregulation was due to the consumption of the metabolic pathways involved.

## 4. Material and Methods 

### 4.1. Semen Collection

**Figure 8:**
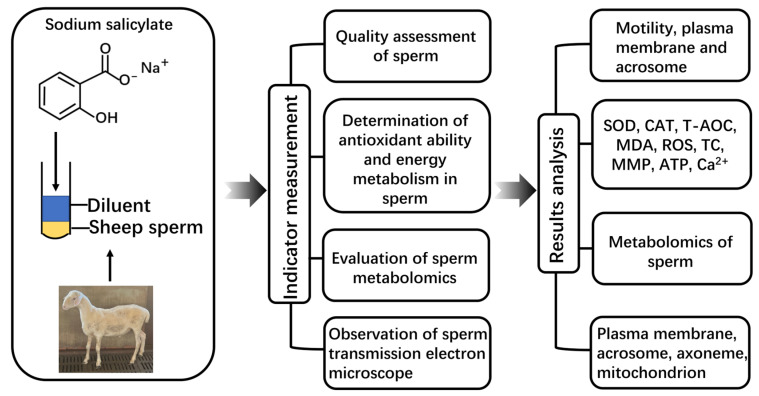
The workflow of the impact of SS on sheep sperm.

This study used an artificial vagina to collect semen from 6 healthy and fertile Hu sheep (aged 3 to 5). Semen from 6 sheep was collected twice a week for six consecutive weeks, and 72 samples were obtained. The computer-assisted sperm analysis (CASA) system (Songjingtianlun Biotechnology, Nanjing, China) was used to evaluate sperm motility. Sperm with motility ≥ 80%, abnormal morphology ≤ 15%, and density ≥ 2.0 × 10^9^ were used for this experiment. The entire experimental design and workflow are shown in Figure 8.

### 4.2. Preparation of Diluent

Unless otherwise specified, all chemicals and reagents used in this study were purchased from Sigma Aldrich (Beijing, China). Every 100 mL of semen diluent contained 1.26 g of fructose, 1.72 g of citric acid monohydrate, 3.53 g of trimethylaminomethane, 3.8 g of vitamin E, 20 mL of fresh egg yolk, 4 mL of glycerol, 15,000 IU of penicillin sodium, 15,000 IU of streptomycin, 0.5 g of vitamin C, and 0.5 g of bovine serum albumin.

### 4.3. Semen Processing

The semen samples collected each time (*n* = 6) were mixed, and the diluent was adjusted to a semen density of 1.0 × 10^8^ spermatozoa/mL. Different concentrations of SS or CC were added to the diluted semen and divided into the following groups: SS control, the 10 μM SS, 20 μM SS, 30 μM SS, and 50 μM SS group (containing 0, 10, 20, 30, 50μM of SS, respectively); the CC control, and the 1 μM CC, 5 μM CC, 10 μM CC, and 20 μM CC group (containing 0, 1, 5, 10, 20 μM of CC, respectively). The samples were wrapped in cotton and stored in a refrigerator at 4 °C.

Five groups of samples treated with SS were stored at 4 °C for 20 days. Sperm motility, plasma membrane, and acrosome integrity were tested every two days to screen for the optimal concentration of SS. Then, on the 16th day, the effects of the optimal concentration of SS on the levels of SOD, CAT, T-AOC, ROS, MDA, MMP, ATP, TC, and Ca^2+^ in the sperm were tested, and an ultrastructural observation and metabolomics analysis was conducted. The five samples processed by CC were used to verify the low-temperature protective effect of the AMPK signaling pathway on sperm. All experiments were independently repeated at least three times.

### 4.4. Sperm Motility

A 10 μL semen sample was taken and placed on a glass slide preheated at 37 °C and covered with a cover glass. The motility was evaluated using a CASA system under a phase contrast microscope (200×).

### 4.5. Plasma Membrane Integrity

This experiment used the hypoosmotic swelling test (HOST) to evaluate the integrity of the sperm plasma membrane. A 20 μL semen sample was mixed with 200 μL of low osmotic solution (0.838 g of sodium citrate and 1.38 g of fructose were dissolved in 100 mL of double distilled water, 150 mOsm), and incubated at 37 °C for 30 min. A 10 μL sample was taken and observed under a phase contrast microscope (400×). The sperm with a curled tail were considered sperm with an intact plasma membrane, and the ratio of sperm with intact plasma membranes was calculated.

### 4.6. Acrosome Integrity

This experiment used the Giemsa staining method to detect the integrity of the acrosome. A 20 μL semen sample was dropped onto a glass slide, smeared and air dried, fixed with 4% formaldehyde for 15 min, washed, and air dried, and then stained with a Giemsa staining solution for 2 h. After washing and air drying, the acrosome was observed under a microscope, and the percentage of intact sperm was calculated.

A 20 µL semen sample was mixed with 200 µL of phosphate-buffered saline (PBS) and centrifuged at 1800 rpm for 3 min. The supernatant was discarded, and the sperm was fixed with 4% paraformaldehyde for 15 min. The supernatant was removed via centrifugation, and 50 µL of PBS and 5 µL of fluorescent isothiocyanate-labeled peanut agglutinin (FITC-PNA) was added to prepare the sperm sample and observed under a fluorescence microscope.

### 4.7. CAT, SOD, TC, MDA, T-AOC, and Ca^2+^ Levels

The CAT assay kit (BC 0205), SOD assay kit (BC 0175), TC assay kit (BC 1980), and MDA assay kit (BC0025) were purchased from Solarbio Science&Technology Co., Ltd. (Beijing, China), T-AOC assay kit (S0121) was purchased from Beyond Biotechnology Co., Ltd. (Shanghai, China), and the Ca^2+^ assay kit (R22060) was purchased from Yuanye Biotechnology Co., Ltd. (Shanghai, China). A 20 µL semen sample was mixed with 1 mL of the extract, and the semen sample was ground with a homogenizer for 5 min. The supernatant was centrifuged, and the levels of CAT, SOD, TC, MDA, T-AOC, and Ca^2+^ were measured using a spectrophotometer or enzyme-linked immunosorbent assay according to the manufacturer’s instructions.

### 4.8. ROS, MMP, and ATP Levels

A ROS detection kit (S0033S) (Beyotime Biotechnology, Shanghai, China) was used to detect sperm ROS levels. DCFH-DA (Fluorescence probe) was diluted with PBS at a ratio of 1:1000, and 1 mL of the dilution was mixed with 20 µL of semen and incubated at 37 °C for 20 min; the fluorescent microplate reader was used to measure the absorbance of the excitation wavelength at 488 nm and emission wavelength at 525 nm.

Sperm MMP levels were evaluated using the MMP detection kit (C2006) (Beyotime Biotechnology, Shanghai, China). Semen was added to PBS, and the sperm density was adjusted to 1 × 10^6^ spermatozoa/mL using PBS. Then, 5 µL of the JC-1 staining solution was added and incubated in the dark at 4 °C for 30 min. The stained sample was filtered using a 300-mesh filter, and the MMP of the sperm was detected using a flow cytometer.

The sperm ATP content was measured using an ATP detection kit (S0026) (Beyond Biotechnology, Shanghai, China). A 20 µL semen sample was taken wherein 1 mL of the cell lysate was added, centrifuged at 4 °C for 10 min, and the supernatant was collected. The ATP content of sperm was analyzed using a fluorescence microplate reader, and the results were calculated based on the standard curve.

### 4.9. Ultrastructural Detection

Sperm was fixed overnight at 4 °C with 2.5% glutaraldehyde, washed with PBS, fixed with 1% osmic acid for 2 h, washed with PBS, dehydrated with concentration gradient ethanol, resin-embedded samples, and the samples were sliced into 70–90 nm sections using an ultra-thin slicing machine. The samples were stained with a lead citrate solution and uranyl acetate solution and observed under transmission electron microscopy.

### 4.10. Metabolomics Analysis

Sheep sperm were divided into an SS control group and a 20 μM SS group. A semen sample was taken from the 16th day of low-temperature preservation, and 1 mL of the extraction solution (methanol/acetonitrile/water = 2:2:1 (*v*/*v*)) was added to the sample. It was frozen in liquid nitrogen for 1 min and then thawed and mixed for 30 s. The sample was sonicated in an ice water bath for 10 min and left to stand at −40 °C for 1 h. The sample was centrifuged at 4 °C and 12,000 rpm for 15 min. The supernatant was taken and placed in an injection bottle for machine testing. The experimental samples were analyzed via LC-MS/MS using a UHPLC system (Vanquish, Thermo Fisher Scientific, Beijing, China), which has a UPLC BEH amide column (2.1 mm × 100 mm, 1.7 μm) coupled with the Thermo Q Exactive HFX mass spectrometer (Orbitrap MS, Thermo Fisher Scientific, Beijing, China). All samples were mixed with an equal amount of supernatant to form a quality control (QC) sample for machine testing, which was used to monitor and evaluate the stability of the system and the reliability of experimental data.

### 4.11. Statistical Analysis

All data were analyzed using SPSS software, version 26.0 (IBMCorp., Released 2019, Armonk, N.Y., USA), and one-way ANOVA was performed on sperm motility, plasma membrane, and acrosome integrity. The results were presented in the form of the mean ± standard deviation. Duncan’s method was used for multiple comparisons, with *p* < 0.05 indicating significant differences and *p* > 0.05 indicating no significant differences. T-tests were conducted on sperms’ antioxidant capacity, mitochondrial activity, Ca^2+^, and TC content and were plotted using GraphPad Prism 8.0.

## 5. Conclusions

**Figure 9:**
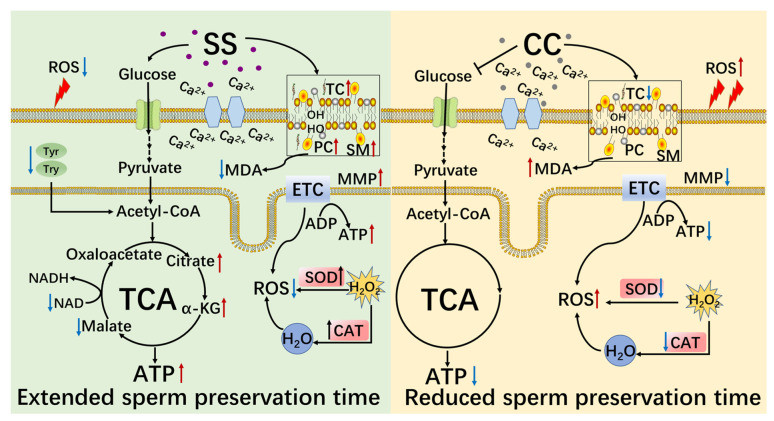
The regulatory mechanism of SS and CC on sheep sperm metabolism. The red arrow indicates an increase; the blue arrow indicates a decrease; black arrows indicate association. ROS, reactive oxygen species; TC, total cholesterol; PC, phosphatidylcholine; SM, sphingomyelin; MMP, mitochondrial membrane potential; ETC, electron transport chain; Tyr, tyrosine; MDA, malondialdehyde; TCA, tricarboxylic acid cycle; SOD, superoxide dismutase; CAT, catalase; ADP, adenosine diphosphate; ATP, adenosine triphosphate; α-KG, α-Ketoglutaric acid; NAD, nicotinamide adenine dinucleotide; NADH, nicotinamide adenine dinucleotide (reducibility).

The results confirmed that under low-temperature conditions, SS prolonged the in vitro preservation time and quality of sheep sperm by enhancing their antioxidant capacity and regulating sperm metabolism. On the contrary, CC reduced the antioxidant capacity of goat sperm, thereby affecting the in vitro storage time and quality of goat sperm (Figure 9).

## Figures and Tables

**Table 1:** Effects of different concentrations of sodium salicylate on the motility, plasma membrane integrity, and acrosome integrity of ram sperm preserved at 4 °C.

Time (d)	Sodium Salicylate Treatment (μM)	Motility (%)	Plasma Membrane Integrity	Acrosome Integrity
1	0	89.68 ± 4.53 ^bc^	0.87 ± 0.03 ^a^	0.90 ± 0.01 ^a^
	10	86.16 ± 1.53 ^bc^	0.85 ± 0.03 ^a^	0.91 ± 0.03 ^a^
	20	90.19 ± 0.80 ^ab^	0.88 ± 0.02 ^a^	0.93 ± 0.02 ^a^
	30	91.65 ± 3.33 ^ab^	0.87 ± 0.02 ^a^	0.92 ± 0.01 ^a^
	50	90.75 ± 2.49 ^ab^	0.87 ± 0.01 ^a^	0.91 ± 0.01 ^a^
2	0	85.32 ± 1.47 ^b^	0.81 ± 0.04 ^c^	0.89 ± 0.04 ^ab^
	10	85.67 ± 1.12 ^b^	0.84 ± 0.01 ^bc^	0.91 ± 0.04 ^ab^
	20	88.68 ± 1.90 ^a^	0.88 ± 0.03 ^a^	0.92 ± 0.02 ^a^
	30	89.44 ± 1.50 ^a^	0.87 ± 0.02 ^a^	0.89 ± 0.03 ^a^
	50	86.88 ± 1.75 ^ab^	0.83 ± 0.04 ^bc^	0.87 ± 0.02 ^b^
4	0	85.25 ± 1.84 ^b^	0.78 ± 0.05 ^b^	0.82 ± 0.02 ^b^
	10	85.48 ± 2.10 ^b^	0.83 ± 0.05 ^ab^	0.87 ± 0.07 ^ab^
	20	87.39 ± 2.07 ^a^	0.86 ± 0.05 ^a^	0.88 ± 0.04 ^a^
	30	88.43 ± 1.75 ^a^	0.86 ± 0.02 ^a^	0.88 ± 0.01 ^ab^
	50	86.28 ± 1.99 ^ab^	0.82 ± 0.02 ^ab^	0.86 ± 0.04 ^ab^
6	0	84.13 ± 1.61 ^b^	0.75 ± 0.03 ^b^	0.78 ± 0.03 ^b^
	10	84.80 ± 1.32 ^b^	0.81 ± 0.08 ^ab^	0.85 ± 0.03 ^a^
	20	86.08 ± 1.75 ^ab^	0.85 ± 0.03 ^a^	0.87 ± 0.02 ^a^
	30	88.03 ± 1.52 ^a^	0.85 ± 0.03 ^a^	0.86 ± 0.02 ^a^
	50	84.75 ± 1.35 ^ab^	0.82 ± 0.02 ^ab^	0.83 ± 0.02 ^a^
8	0	83.01 ± 1.94 ^b^	0.71 ± 0.14 ^b^	0.71 ± 0.05 ^b^
	10	83.13 ± 2.28 ^b^	0.80 ± 0.02 ^ab^	0.80 ± 0.03 ^ab^
	20	85.55 ± 1.91 ^a^	0.83 ± 0.05 ^ab^	0.83 ± 0.06 ^a^
	30	87.03 ± 1.12 ^a^	0.84 ± 0.02 ^a^	0.82 ± 0.04 ^a^
	50	84.59 ± 1.39 ^b^	0.81 ± 0.04 ^ab^	0.81 ± 0.05 ^a^
10	0	79.07 ± 1.74 ^c^	0.67 ± 0.03 ^b^	0.63 ± 0.03 ^d^
	10	82.00 ± 1.80 ^b^	0.78 ± 0.03 ^a^	0.80 ± 0.05 ^ab^
	20	85.06 ± 2.06 ^ab^	0.80 ± 0.05 ^a^	0.81 ± 0.02 ^a^
	30	81.60 ± 1.95 ^b^	0.79 ± 0.06 ^a^	0.77 ± 0.04 ^ab^
	50	81.26 ± 1.44 ^bc^	0.77 ± 0.04 ^a^	0.72 ± 0.04 ^b^
12	0	72.79 ± 1.76 ^c^	0.62 ± 0.08 ^c^	0.60 ± 0.04 ^d^
	10	76.26 ± 1.83 ^b^	0.72 ± 0.02 ^b^	0.75 ± 0.03 ^ab^
	20	80.77 ± 2.56 ^a^	0.78 ± 0.04 ^a^	0.79 ± 0.04 ^a^
	30	80.49 ± 1.31 ^a^	0.76 ± 0.04 ^a^	0.76 ± 0.06 ^a^
	50	77.89 ± 2.53 ^ab^	0.75 ± 0.07 ^a^	0.69 ± 0.05 ^c^
14	0	66.15 ± 1.50 ^d^	0.56 ± 0.06 ^b^	0.54 ± 0.06 ^c^
	10	70.42 ± 1.38 ^c^	0.72 ± 0.06 ^a^	0.70 ± 0.16 ^ab^
	20	79.25 ± 1.67 ^a^	0.75 ± 0.11 ^a^	0.76 ± 0.01 ^a^
	30	78.99 ± 1.24 ^a^	0.73 ± 0.02 ^a^	0.74 ± 0.04 ^a^
	50	75.95 ± 2.06 ^b^	0.59 ± 0.05 ^b^	0.67 ± 0.13 ^b^
16	0	55.01 ± 3.70 ^d^	0.52 ± 0.05 ^c^	0.47 ± 0.02 ^c^
	10	64.32 ± 1.70 ^b^	0.64 ± 0.05 ^a^	0.60 ± 0.09 ^b^
	20	71.46 ± 1.80 ^a^	0.68 ± 0.08 ^a^	0.71 ± 0.02 ^a^
	30	69.28 ± 2.00 ^a^	0.63 ± 0.11 ^b^	0.67 ± 0.05 ^ab^
	50	60.42 ± 2.11 ^c^	0.57 ± 0.07 ^c^	0.63 ± 0.03 ^b^
18	0	52.91 ± 1.27 ^c^	0.47 ± 0.03 ^c^	0.42 ± 0.05 ^b^
	10	60.51 ± 2.92 ^b^	0.58 ± 0.05 ^a^	0.58 ± 0.06 ^a^
	20	68.27 ± 2.34 ^a^	0.61 ± 0.09 ^a^	0.66 ± 0.03 ^a^
	30	65.18 ± 2.18 ^b^	0.60 ± 0.07 ^a^	0.60 ± 0.15 ^a^
	50	51.64 ± 2.34 ^c^	0.56 ± 0.03 ^b^	0.59 ± 0.11 ^a^
20	0	43.41 ± 1.69 ^e^	0.42 ± 0.04 ^c^	0.39 ± 0.05 ^c^
	10	55.75 ± 1.61 ^c^	0.54 ± 0.06 ^a^	0.49 ± 0.05 ^b^
	20	62.80 ± 2.29 ^a^	0.59 ± 0.05 ^a^	0.57 ± 0.05 ^a^
	30	59.43 ± 1.50 ^b^	0.56 ± 0.06 ^a^	0.54 ± 0.03 ^a^
	50	51.33 ± 1.24 ^d^	0.51 ± 0.07 ^b^	0.50 ± 0.06 ^b^

Values denote the mean ± standard deviation. In the same column with the same preservation time, different letters indicate significant differences (*p* < 0.05).

**Table 2:** Effects of different concentrations of compound C on the motility, plasma membrane integrity, and acrosome integrity of ram sperm preserved at 4 °C.

Time (d)	Compound C Treatment (μM)	Motility (%)	Plasma Membrane Integrity	Acrosome
1	0	88.33 ± 0.94 ^d^	0.86 ± 0.02 ^a^	0.92 ± 0.02 ^ab^
	1	93.69 ± 0.74 ^a^	0.90 ± 0.05 ^a^	0.93 ± 0.01 ^a^
	5	90.89 ± 1.56 ^bc^	0.89 ± 0.02 ^a^	0.92 ± 0.02 ^ab^
	10	91.71 ± 1.31 ^ab^	0.87 ± 0.04 ^a^	0.91 ± 0.02 ^ab^
	20	88.90 ± 2.39 ^cd^	0.84 ± 0.02 ^a^	0.90 ± 0.01 ^b^
2	0	87.68 ± 1.57 ^a^	0.87 ± 0.03 ^a^	0.91 ± 0.00 ^a^
	1	90.39 ± 1.57 ^a^	0.86 ± 0.03 ^a^	0.91 ± 0.01 ^a^
	5	87.14 ± 0.99 ^a^	0.85 ± 0.04 ^a^	0.90 ± 0.01 ^a^
	10	88.91 ± 1.67 ^a^	0.85 ± 0.01 ^a^	0.90 ± 0.01 ^a^
	20	87.20 ± 1.22 ^a^	0.84 ± 0.03 ^a^	0.90 ± 0.01 ^a^
3	0	86.29 ± 2.00 ^a^	0.85 ± 0.04 ^a^	0.90 ± 0.01 ^a^
	1	85.84 ± 2.01 ^a^	0.85 ± 0.05 ^a^	0.89 ± 0.01 ^a^
	5	85.55 ± 1.11 ^a^	0.84 ± 0.03 ^a^	0.88 ± 0.01 ^a^
	10	85.43 ± 2.38 ^a^	0.82 ± 0.03 ^a^	0.88 ± 0.03 ^a^
	20	82.85 ± 0.72 ^b^	0.81 ± 0.07 ^a^	0.85 ± 0.01 ^b^
4	0	85.64 ± 1.27 ^a^	0.81 ± 0.01 ^a^	0.86 ± 0.02 ^a^
	1	84.88 ± 2.76 ^a^	0.80 ± 0.01 ^a^	0.85 ± 0.01 ^a^
	5	84.18 ± 1.52 ^a^	0.79 ± 0.05 ^a^	0.82 ± 0.02 ^b^
	10	82.31 ± 1.96 ^ab^	0.78 ± 0.03 ^a^	0.79 ± 0.01 ^c^
	20	79.73 ± 2.13 ^b^	0.76 ± 0.03 ^ab^	0.76 ± 0.02 ^d^
5	0	84.35 ± 1.02 ^a^	0.79 ± 0.02 ^a^	0.82 ± 0.01 ^a^
	1	80.31 ± 1.04 ^ab^	0.77 ± 0.03 ^ab^	0.77 ± 0.02 ^b^
	5	79.79 ± 5.48 ^ab^	0.73 ± 0.05 ^bc^	0.77 ± 0.03 ^b^
	10	78.10 ± 2.92 ^b^	0.73 ± 0.01 ^bc^	0.76 ± 0.02 ^bc^
	20	77.24 ± 1.38 ^b^	0.71 ± 0.02 ^c^	0.73 ± 0.01 ^c^
6	0	81.66 ± 1.41 ^a^	0.78 ± 0.02 ^a^	0.80 ± 0.01 ^a^
	1	76.08 ± 1.25 ^b^	0.72 ± 0.01 ^b^	0.73 ± 0.02 ^b^
	5	74.49 ± 2.06 ^b^	0.69 ± 0.04 ^bc^	0.71 ± 0.03 ^b^
	10	71.30 ± 2.52 ^c^	0.67 ± 0.04 ^bc^	0.71 ± 0.03 ^b^
	20	69.70 ± 1.46 ^c^	0.63 ± 0.05 ^c^	0.66 ± 0.03 ^c^
7	0	79.32 ± 1.87 ^a^	0.77 ± 0.06 ^a^	0.77 ± 0.04 ^a^
	1	67.71 ± 1.75 ^b^	0.66 ± 0.02 ^b^	0.71 ± 0.03 ^b^
	5	65.61 ± 0.93 ^b^	0.64 ± 0.02 ^b^	0.67 ± 0.04 ^bc^
	10	64.99 ± 3.91 ^b^	0.63 ± 0.05 ^b^	0.62 ± 0.01 ^cd^
	20	57.76 ± 2.50 ^c^	0.54 ± 0.01 ^c^	0.59 ± 0.02 ^d^

Values denote the mean ± standard deviation. In the same column with the same preservation time, different letters indicate significant differences (*p* < 0.05).

**Table 3:** Analysis of the metabolic pathway of sheep sperm in sodium salicylate group.

Pathway	Total	Hits	Raw p	Impact	Hits ID
Nitrogen metabolism	9	2	0.033226	0	L-Glutamine, L-Histidine
Histidine metabolism	14	2	0.075649	0.26619	L-Histidine, L-Aspartic acid
Glyoxylate and dicarboxylate metabolism	16	2	0.095705	0.33334	Glycolic acid, citric acid
Phenylalanine, tyrosine and tryptophan biosynthesis	4	1	0.12599	0.5	L-Phenylalanine
Alanine, aspartate and glutamate metabolism	23	2	0.17481	0.27667	L-Aspartic acid, L-Glutamine
Phenylalanine metabolism	9	1	0.26181	0.40741	L-Phenylalanine

Total, the total number of compounds in the pathway; Hits, the actually matched number from the user uploaded data; Raw p, the original *p*-value calculated from the enrichment analysis; Impact, the pathway impact value calculated from pathway topology analysis; Hits ID, the names of differential metabolites.

## Data Availability

The data supporting this article are available in the article and in its online Supplementary Material.

## Supplementary Materials

The following supporting information can be downloaded at: https://www.mdpi.com/article/10.3390/molecules29010188/s1, Table S1. Differential metabolites between the control group and the sodium salicylate group. Table S2. Enrichment analysis of differential metabolites in sheep sperm from sodium salicylate group and control group. Table S3. Analysis of metabolomic pathways in the control group and sodium salicylate group. Figure S1. Hierarchical cluster analysis thermogram of differential metabolites in sodium salicylate (SS) sheep sperm of the control group.

## References

[1] Salimi A., Eslami M., Farrokhi-Ardabili F. (2023). Influence of trans-ferulic acid on the quality of ram semen upon cold preservation. Vet. Med. Sci..

[2] White I.G. (1993). Lipids and calcium uptake of sperm in relation to cold shock and preservation: A review. Reprod. Fertil. Dev..

[3] Arav A., Pearl M., Zeron Y. (2000). Does lipid profile explain chilling sensitivity and membrane lipid phase transition of spermatozoa and oocytes?. Cryo Lett..

[4] Selvaraju S., Raju P., Rao S.B.N., Raghavendra S., Nandi S., Dineshkumar D., Thayakumar A., Parthipan S., Ravindra J.P. (2012). Evaluation of maize grain and polyunsaturated fatty acid (PUFA) as energy sources for breeding rams based on hormonal, sperm functional parameters and fertility. Reprod. Fertil. Dev..

[5] Taylor C.T. (2001). Antioxidants and reactive oxygen species in human fertility. Environ. Toxicol. Pharmacol..

[6] Agarwal A., Majzoub A. (2017). Role of Antioxidants in Assisted Reproductive Techniques. World J. Men’s Health.

[7] Agarwal A., Saleh R.A., Bedaiwy M.A. (2003). Role of reactive oxygen species in the pathophysiology of human reproduction. Fertil. Steril..

[8] Allai L., Benmoula A., Maia M.d.S., Nasser B., El Amiri B. (2018). Supplementation of ram semen extender to improve seminal quality and fertility rate. Anim. Reprod. Sci..

[9] Aitken R.J. (1999). The Amoroso Lecture. The human spermatozoon—A cell in crisis?. J. Reprod. Fertil..

[10] Al-Mutary M.G., Al-Ghadi M.Q., Ammari A.A., Al-Himadi A.R., Al-Jolimeed A.H., Arafah M.W., Amran R.A., Aleissa M.S., Swelum A.A.-A. (2020). Effect of different concentrations of resveratrol on the quality and in vitro fertilizing ability of ram semen stored at 5 degrees C for up to 168 h. Theriogenology.

[11] Zhang X.-G., Liu Q., Wang L.-Q., Yang G.-S., Hu J.-H. (2016). Effects of glutathione on sperm quality during liquid storage in boars. Anim. Sci. J..

[12] Madunic J., Horvat L., Majstorovic I., Jodlowska I., Antica M., Matulic M. (2017). Sodium Salicylate Inhibits Urokinase Activity in MDA MB-231 Breast Cancer Cells. Clin. Breast Cancer.

[13] Madathil S.K., Karuppagounder S.S., Mohanakumar K.P. (2013). Sodium salicylate protects against rotenone-induced Parkinsonism in rats. Synapse.

[14] Colantoni A., de Maria N., Caraceni P., Bernardi M., Floyd R.A., Van Thiel D.H. (1998). Prevention of reoxygenation injury by sodium salicylate in isolated-perfused rat liver. Free Radic. Biol. Med..

[15] Vincent E.E., Coelho P.P., Blagih J., Griss T., Viollet B., Jones R.G. (2015). Differential effects of AMPK agonists on cell growth and metabolism. Oncogene.

[16] Herzig S., Shaw R.J. (2018). AMPK: Guardian of metabolism and mitochondrial homeostasis. Nat. Rev. Mol. Cell Biol..

[17] Martin-Hidalgo D., Hurtado de Llera A., Calle-Guisado V., Gonzalez-Fernandez L., Garcia-Marin L., Julia Bragado M. (2018). AMPK Function in Mammalian Spermatozoa. Int. J. Mol. Sci..

[18] Thi Mong Diep N., Froment P., Combarnous Y., Blesbois E. (2016). AMPK, regulator of sperm energy and functions. Med. Sci. M/S.

[19] Dasgupta B., Seibel W. (2018). Compound C/Dorsomorphin: Its Use and Misuse as an AMPK Inhibitor. Methods Mol. Biol..

[20] Jin J., Mullen T.D., Hou Q., Bielawski J., Bielawska A., Zhang X., Obeid L.M., Hannun Y.A., Hsu Y.-T. (2009). AMPK inhibitor Compound C stimulates ceramide production and promotes Bax redistribution and apoptosis in MCF7 breast carcinoma cells. J. Lipid Res..

[21] Amann R., Peskar B.A. (2002). Anti-inflammatory effects of aspirin and sodium salicylate. Eur. J. Pharmacol..

[22] Kotschwar J.L., Coetzee J.F., Anderson D.E., Gehring R., KuKanich B., Apley M.D. (2009). Analgesic efficacy of sodium salicylate in an amphotericin B-induced bovine synovitis-arthritis model. J. Dairy Sci..

[23] Kopp E., Ghosh S. (1994). Inhibition of NF-kappa B by sodium salicylate and aspirin. Science.

[24] Steinberg G.R., Dandapani M., Hardie D.G. (2013). AMPK: Mediating the metabolic effects of salicylate-based drugs?. Trends Endocrinol. Metab..

[25] Hawley S.A., Fullerton M.D., Ross F.A., Schertzer J.D., Chevtzoff C., Walker K.J., Peggie M.W., Zibrova D., Green K.A., Mustard K.J. (2012). The Ancient Drug Salicylate Directly Activates AMP-Activated Protein Kinase. Science.

[26] Kimura K. (1997). Mechanisms of active oxygen species reduction by non-steroidal anti-inflammatory drugs. Int. J. Biochem. Cell Biol..

[27] Piomboni P., Focarelli R., Stendardi A., Ferramosca A., Zara V. (2012). The role of mitochondria in energy production for human sperm motility. Int. J. Androl..

[28] Shi L., Zhang Y., Huang X., Shi M., Sun D., Zhang Y., Li W., Jin T., Feng J., Xing J. (2022). Effects of mitoquinone (MitoQ) supplementation during boar semen cryopreservation on sperm quality, antioxidant status and mitochondrial proteomics. Anim. Reprod. Sci..

[29] Lan Q., Xue L.E., Cao J., Xie Y., Xiao T., Fang S. (2022). Caffeic Acid Phenethyl Ester (CAPE) Improves Boar Sperm Quality and Antioxidant Capacity in Liquid Preservation (17 degrees C) Linked to AMPK Activity Maintenance. Front. Vet. Sci..

[30] Aurich C. (2005). Factors affecting the plasma membrane function of cooled-stored stallion spermatozoa. Anim. Reprod. Sci..

[31] Moce E., Blanch E., Tomas C., Graham J.K. (2010). Use of Cholesterol in Sperm Cryopreservation: Present Moment and Perspectives to Future. Reprod. Domest. Anim..

[32] Parks J.E., Graham J.K. (1992). Effects of cryopreservation procedures on sperm membranes. Theriogenology.

[33] Yang S.X., Adams G.P., Zwiefelhofer E.M., Rajapaksha K., Anzar M. (2021). Cholesterol-cyclodextrin complex as a replacement for egg yolk in bull semen extender: Sperm characteristics post-thawing and in vivo fertility. Anim. Reprod. Sci..

[34] Hurtado de Llera A., Martin-Hidalgo D., Rodriguez-Gil J.E., Cruz Gil M., Garcia-Marin L.J., Julia Bragado M. (2013). AMP-activated kinase, AMPK, is involved in the maintenance of plasma membrane organization in boar spermatozoa. Biochim. Biophys. Acta-Biomembr..

[35] Lindemann C.B., Lesich K.A. (2016). Functional anatomy of the mammalian sperm flagellum. Cytoskeleton.

[36] Lesich K.A., Kelsch C.B., Ponichter K.L., Dionne B.J., Dang L., Lindemann C.B. (2012). The Calcium Response of Mouse Sperm Flagella: Role of Calcium Ions in the Regulation of Dynein Activity. Biol. Reprod..

[37] Eddy E.M., Toshimori K., O’Brien D.A. (2003). Fibrous sheath of mammalian spermatozoa. Microsc. Res. Tech..

[38] Krisfalusi M., Miki K., Magyar P.L., O’Brien D.A. (2006). Multiple glycolytic enzymes are tightly bound to the fibrous sheath of mouse spermatozoa. Biol. Reprod..

[39] Amores-Sanchez M.I., Medina M.A. (1999). Glutamine, as a precursor of glutathione, and oxidative stress. Mol. Genet. Metab..

[40] Zhu Z., Fan X., Lv Y., Lin Y., Wu D., Zeng W. (2017). Glutamine protects rabbit spermatozoa against oxidative stress via glutathione synthesis during cryopreservation. Reprod. Fertil. Dev..

[41] Wang S., Sun M., Wang N., Yang K., Guo H., Wang J., Zhang Y., Yue S., Zhou J. (2018). Effects of L-glutamine on boar sperm quality during liquid storage at 17 degrees C. Anim. Reprod. Sci..

[42] Miao Y., Cui Z., Gao Q., Rui R., Xiong B. (2020). Nicotinamide Mononucleotide Supplementation Reverses the Declining Quality of Maternally Aged Oocytes. Cell Rep..

[43] Di Emidio G., Falone S., Artini P.G., Amicarelli F., D’Alessandro A.M., Tatone C. (2021). Mitochondrial Sirtuins in Reproduction. Antioxidants.

[44] Hopp A.-K., Grueter P., Hottiger M.O. (2019). Regulation of Glucose Metabolism by NAD(+) and ADP-Ribosylation. Cells.

