# Supplementary material for: Metabolomics Analysis of Sodium Salicylate Improving the Preservation Quality of Ram Sperm
Source: Molecules. 2023 Dec 28;29(1):188. doi: 10.3390/molecules29010188 (PMC10780297; doi:10.3390/molecules29010188)
Supplement: Supplementary file 1 [file molecules-29-00188-s001.zip › molecules-2783354-supplementary.pdf]

Table S1. Differential metabolites between the control group and the sodium salicylate group.

Name	RT	MZ	VIP	*p*-value	FC
Oxalic acid	431.894	88.98709	1.375965	0.014178	0.847817
L-Threonic acid	323.112	135.029	1.62059	0.002207	1.50154
Pentanenitrile	273.6	84.08132	1.311646	0.036904	0.669882
2-Ketobutyric acid	400.775	101.0234	1.425577	0.011126	1.132806
Glycolic acid	66.8704	75.00778	1.351102	0.017168	0.813339
Glyceraldehyde	289.1505	89.02344	1.242214	0.02418	1.155857
Adenosine	172.648	268.1035	1.348196	0.016942	1.395167
Acetylhydrazine	505.37	75.05595	1.484944	0.009333	0.556198
benzene-1,2,4-triol	372.147	125.0235	1.437455	0.008445	1.20326
Citric acid	488.522	191.0189	1.290371	0.031218	1.509308
4-(Trimethylammonio)butanoate	395.862	146.1175	1.634084	0.003163	1.177757
Terephthalic acid	377.2445	165.0186	1.542464	0.002478	0.868392
Hypoxanthine	171.511	137.0456	1.387601	0.028497	0.917973
Metenamine	283.654	141.1133	1.836486	0.000352	0.681416
2-Keto-3-deoxy-D-gluconic acid	43.8119	177.0398	1.287198	0.032853	0.717181
Gentisaldehyde	35.6681	137.0235	2.223218	8.52E-08	0.005565
(Â±)-Camphoric acid	245.354	201.1118	1.175752	0.036512	1.091553
L-Phenylalanine	276.246	164.071	1.449835	0.00625	1.529529
3-Hydroxycinnamic acid	49.4764	163.0393	1.096695	0.042245	1.21376
L-Gulose	358.2965	179.0554	1.26942	0.023924	1.238461
L-Malic acid	423.4315	133.0134	1.523123	0.005607	1.616739
L-Histidine	415.859	156.0768	1.476974	0.011525	1.364208
Pyroglutamic acid	399.171	130.0499	1.449978	0.013113	1.281121
2-Furoic acid	488.5485	111.0078	1.30652	0.032348	1.520526
Phenylpyruvic acid	36.80135	163.0394	1.76049	0.000187	1.46327
DL-Tyrosine	320.701	182.081	1.53939	0.004665	1.575195
Sorbitol	304.298	181.071	1.580987	0.005044	1.308633
Prostaglandin D1	94.2597	353.2334	1.404451	0.012997	1.27077
Hydroxyprolyl-Hydroxyproline	434.733	245.113	1.492353	0.006772	1.433939
Graveoline	392.405	280.0916	1.377142	0.009047	1.346886
Leucinic acid	111.476	131.0704	1.403152	0.014514	1.203754
Sphinganine	108.8	302.3049	1.397293	0.010263	0.705231
2-Methylbutyroylcarnitine	260.734	246.1696	1.769086	0.000719	1.21192
Pyro-L-glutaminyl-L-glutamine	348.6	258.1081	1.225077	0.037874	1.16117
L-Glutamine	391.886	145.061	1.6974	0.00113	1.284375
Methylmalonic acid	192.722	117.0183	1.470976	0.00852	0.826411
L-Gulonolactone	66.8704	177.0398	1.382688	0.016748	0.846332
Cinnamylideneacetone	187.8215	173.0918	1.307632	0.010948	1.206432
3-[4-Hydroxy-3-(3-methyl-2-butenyl)phenyl]-2-propenal	358.5285	217.1181	1.135966	0.032392	1.143857
5-Hydroxytryptophan	204.16	265.0854	1.321379	0.03491	0.82637
15-KETE	94.2712	317.2123	1.442439	0.007558	1.266711

Glucosamine	173.7475	192.1228	1.323007	0.012413	1.186342
L-trans-4-Methyl-2-pyrrolidinecarboxylic acid	544.065	130.0863	1.573327	0.005448	1.513784
2-Furoylglycine	415.9395	168.0271	1.159341	0.006527	1.542255
6-Ketoprostaglandin E1	218.637	367.2127	1.357169	0.016179	1.116002
Prolyl-Glutamine	381.744	244.129	1.255068	0.028791	1.224466
Dihydrolipoate	66.8376	207.0505	1.422022	0.015441	0.790954
Thiabendazole	478.077	202.0472	1.710373	0.000806	2.013532
NAD	456.405	664.116	1.414466	0.000671	2.395318
O-Acetyl-L-serine	416.651	148.0603	1.262936	0.044047	1.254757
(E)-2-Tridecene-4,6,8-triyn-1-ol	351.534	187.1075	1.382798	0.013647	1.340684
Hippuric acid	203.31	178.0503	1.253633	0.01818	1.155507
D-Xylulose	270.638	149.0447	1.418421	0.009965	1.210459
N-a-Acetyl-L-arginine	389.945	217.1292	1.315132	0.016425	1.242728
14,19-Didehydrocondyfolan	353.938	267.1911	1.373135	0.013169	1.347249
PC(18:3(6Z,9Z,12Z)/P-18:1(11Z))	130.202	766.5744	1.518575	0.005996	1.49453
alpha-Tocopherol acetate	84.6034	473.3985	1.360848	0.024393	0.714723
Cytarabine	407.484	244.0945	1.18628	0.04171	0.879318
Dehydroascorbic acid	109.166	173.0084	1.627032	0.005286	1.727105
Benzoylformic acid	134.473	149.027	1.11104	0.044793	0.854177
PC(22:1(13Z)/14:0)	160.8305	788.6149	1.202722	0.036546	0.641904
PC(16:1(9Z)/P-18:1(11Z))	142.854	742.5733	1.327208	0.027121	1.350365
Mukurozidiol	111.667	335.1055	1.511255	0.004464	1.329536
Taurocholic acid	205.84	514.2847	1.125268	0.027323	1.224036
PC(16:1(9Z)/14:0)	158.7835	704.5215	1.479914	0.008002	1.262633
3-Hydroxymethylglutaric acid	268.75	163.0598	1.185075	0.03011	1.45394
Dimethyl dialkyl ammonium chloride	113.186	304.2994	1.884476	0.003051	0.36095
SM(d17:1/24:1(15Z))	196.079	799.6677	1.371156	0.004481	0.603633
PC(14:0/14:0)	162.3435	678.5064	1.314746	0.037879	1.285886
PC(18:1(9Z)/18:0)	281.426	788.6159	1.14331	0.041971	0.76212
PC(18:4(6Z,9Z,12Z,15Z)/18:2(9Z,12Z))	144.046	778.5372	1.426958	0.007028	1.490229
PC(20:5(5Z,8Z,11Z,14Z,17Z)/P-18:1(11Z))	63.96045	790.5732	1.343484	0.036494	1.415844
SM(d18:1/12:0)	206.713	647.5121	1.278955	0.035353	1.302706
2-Methyl-4-oxopentanedioic acid	68.51625	161.0444	1.156154	0.029609	0.746379
PC(18:1(11Z)/15:0)	92.4771	746.5707	1.739019	0.005301	0.536267
PC(P-18:1(11Z)/20:5(5Z,8Z,11Z,14Z,17Z))	51.2496	790.5735	1.286485	0.037155	1.369532
PC(P-18:1(11Z)/22:6(4Z,7Z,10Z,13Z,16Z,19Z))	121.012	816.5898	1.353268	0.027864	1.27622
PC(22:1(13Z)/15:0)	143.688	802.6323	1.563461	0.001468	0.580843
DL-Tryptophan	279.921	203.082	1.615507	0.001678	1.701489
SM(d18:1/24:1(15Z))	195.629	813.6841	1.747175	0.000565	0.531798
SM(d18:1/20:0)	198.064	759.637	1.964901	1.63E-05	0.78075

stearoyl sphingomyelin	200.409	731.6058	1.844195	7.81E-05	0.65732
O-Phosphotyrosine	506.683	262.0503	1.369717	0.019628	1.530632
3-Methylhistidine	398.463	170.0922	1.401868	0.01627	1.278291
Proline betaine	376.97	144.1019	1.684119	0.002407	1.197387
5,7alpha-Dihydro-1,4,4,7a-tetramethyl-4H-indene	32.7231	175.148	1.682699	0.003753	0.589416
Glutaric acid	171.218	131.0341	1.615612	0.005351	0.83757
Tryptophol [xylosyl-(1->6)-glucoside]	224.361	456.1797	1.394924	0.015948	1.115142
Pelargonic acid	42.6326	157.1226	1.331023	0.023555	0.801432
2,3,5-Trimethyl-6-[4-(methylthio)butyl]pyrazine	392.234	225.1441	1.64827	0.019384	3.092337
1-Deoxynojirimycin	185.1205	164.0916	1.385807	0.007089	1.229546
Leukotriene B4	94.2777	335.2228	1.409048	0.0145	1.266647
PC(18:2(9Z,12Z)/15:0)	92.47325	744.5538	1.381948	0.028426	0.581074
Eicosadienoic acid	400.542	292.9981	1.396775	0.010815	0.823322
11,12-Dimethoxydihydrokawain	207.7595	293.1338	1.512027	0.005517	0.668055
Prostaglandin F1a	186.114	355.249	1.329647	0.01928	1.129651
L-Galactose	270.18	198.0969	1.41196	0.016059	1.359687
7-Methylxanthine	338.143	165.0397	1.31565	0.020648	1.235843
Rutacultin	164.7465	275.1233	1.272747	0.046331	0.613272
Phenylalanyl-Arginine	333.296	322.1871	1.20078	0.028843	1.339251
Calystegine B5	317.277	176.0916	1.339174	0.024022	0.663182
Citramalic acid	410.483	147.029	1.346558	0.016418	0.842059
L-Aspartic acid	423.432	132.0294	1.407851	0.010238	1.351397
4-(2,6,6-Trimethyl-1,3-cyclohexadien-1-yl)-2-butanone	32.73725	193.1586	1.543365	0.003957	0.583827
Argininic acid	382.675	176.1029	1.273096	0.010782	1.288826
4-Aminohippuric acid	205.809	193.0613	1.246869	0.021341	1.21141
L-beta-aspartyl-L-threonine	451.5375	235.0921	1.315966	0.043455	0.827499
LysoPC(16:0)	217.063	496.3395	1.166903	0.030179	1.155208
2-Phenylglycine	250.1565	152.0703	1.264468	0.022068	0.75539
12-Oxo-2,3-dinor-10,15-phytodienoic acid	314.8175	265.1754	1.882098	0.00027	1.300812
Glycyl-glycine	400.565	133.0607	1.506078	0.00818	1.212506
1-Isothiocyanato-7-(methylthio)heptane	166.9145	204.0866	1.776403	0.001143	0.767001
2-Oxo-4-methylthiobutanoic acid	57.6908	147.0113	1.398369	0.023504	1.379668
Isoferulic acid 3-sulfate	488.5435	275.0243	1.289441	0.038723	2.497786
4-Amino-2-methylenebutanoic acid	111.76	116.0707	1.411157	0.030057	0.713497
3-Methylglutarylcarnitine	415.64	290.1596	1.326155	0.046555	1.212918
6-Hydroxyshogaol	381.477	293.1703	1.541196	0.007207	1.244805
Cellulose triacetate	476.2	537.2225	2.022396	2.14E-05	2.335078
Mesylate	108.025	94.97987	1.262923	0.030395	0.825107
N,N-Dimethylguanosine	199.9985	310.1147	1.566441	0.004028	1.421784
N6-Acetyl-5S-hydroxy-L-lysine	199.396	205.1181	1.118146	0.042531	1.372259

Achillicin	360.487	307.1473	1.203279	0.037986	1.267438
O-Acetylserine	76.0376	146.045	1.313688	0.031019	0.824637
4-(Methylthio)-1-butanol	117.656	121.0689	1.0519	0.039552	0.661301
Grevilline C	457.385	357.0554	1.229159	0.046031	1.406458
PE(20:2(11Z,14Z)/14:0)	156.6	716.5212	1.301143	0.026592	0.844436
3,4-Dihydroxy-2-hydroxymethyl-1-pyrrolidinepropanamide	187.819	205.1181	1.548526	0.01081	0.568822
Linamarin	262.795	265.1389	1.361727	0.024345	1.206979
Anaxagoreine	195.785	284.1336	1.715487	0.000494	1.401815
1-Deoxy-D-xylulose 5-phosphate	65.8809	213.0167	1.526479	0.015829	0.668049
Valyl-Glutamine	362.75	246.1445	1.39311	0.018079	1.33339
(6R,7S)-6,7-Epoxy-1,3-tetradecadiyne	343.635	205.1546	1.399454	0.006323	0.761678
Valyl-Lysine	557.574	246.181	1.250324	0.022751	1.162855
Nandrolone	88.508	275.1964	1.421584	0.008038	0.598151
N-Acetyl-a-neuraminic acid	201.363	310.1127	1.347094	0.032305	1.642667
6,7-Dihydro-4-(hydroxymethyl)-2-(p-hydroxyphenethyl)-7-methyl-5H-2-pyrindinium	361.174	285.1655	1.351655	0.024334	1.47021
Camelinin	420.127	262.1279	1.951566	0.000156	1.545112
PE(22:2(13Z,16Z)/14:0)	174.3255	744.5536	1.294233	0.024496	0.532996

RT, the chromatographic retention time of the substance; MZ, the mass to charge ratio of characteristic ions in a substance; VIP, variable importance in projection; *p*-value, obtained from the t-test of the substance in this group comparison; FC, fold change.

Table S2. Enrichment analysis of differential metabolites in sheep sperm from sodium salicylate group and control group.

Name	Number	*p*-value	Name	Number	*p*-value
Metabolic pathways	37	0.001018	Taurine and hypotaurine metabolism	1	0.197025
Biosynthesis of amino acids	7	0.000163	Glutathione metabolism	1	0.304535
Biosynthesis of cofactors	7	0.032225	Amino sugar and nucleotide sugar metabolism	1	0.67968
ABC transporters	6	0.001687	Glycosylphosphatidylinositol (GPI)-anchor biosynthesis	1	0.037362
Cysteine and methionine metabolism	5	0.000347	Linoleic acid metabolism	1	0.234549
D-Amino acid metabolism	5	0.000372	alpha-Linolenic acid metabolism	1	0.343488
2-Oxocarboxylic acid metabolism	5	0.008072	Vitamin B6 metabolism	1	0.241846
Central carbon metabolism in cancer	5	2.07E-05	Pantothenate and CoA biosynthesis	1	0.249075
Purine metabolism	4	0.013635	Lipoic acid metabolism	1	0.116516
Arachidonic acid metabolism	4	0.005183	Terpenoid backbone biosynthesis	1	0.355993
Glyoxylate and dicarboxylate metabolism	4	0.002599	Nitrogen metabolism	1	0.165719
Aminoacyl-tRNA biosynthesis	4	0.001451	Sulfur metabolism	1	0.270361
Carbon metabolism	4	0.021833	Drug metabolism - other enzymes	1	0.392129
Protein digestion and absorption	4	0.000921	Biosynthesis of unsaturated fatty acids	1	0.508468
Ascorbate and aldarate metabolism	3	0.016197	Biosynthesis of nucleotide sugars	1	0.857525
Alanine, aspartate and glutamate metabolism	3	0.002192	PPAR signaling pathway	1	0.046488
Histidine metabolism	3	0.009581	cGMP-PKG signaling pathway	1	0.090864
Glycerophospholipid metabolism	3	0.015445	cAMP signaling pathway	1	0.212245
Sphingolipid signaling pathway	3	0.000332	Sulfur relay system	1	0.099495
Neuroactive ligand-receptor interaction	3	0.012639	Autophagy - other	1	0.028151
Galactose metabolism	2	0.069733	Autophagy - animal	1	0.055529
Arginine biosynthesis	2	0.019528	AMPK signaling pathway	1	0.189308
Pyrimidine metabolism	2	0.122297	Longevity regulating pathway	1	0.073361
Glycine, serine and threonine metabolism	2	0.075113	Necroptosis	1	0.090864

beta-Alanine metabolism	2	0.036292	Vascular smooth muscle contraction	1	0.141462
Sphingolipid metabolism	2	0.022881	Glutamatergic synapse	1	0.073361
Propanoate metabolism	2	0.054439	Serotonergic synapse	1	0.330746
Thiamine metabolism	2	0.034226	GABAergic synapse	1	0.082153
Nicotinate and nicotinamide metabolism	2	0.094922	Taste transduction	1	0.263332
Retrograde endocannabinoid signaling	2	0.013517	Inflammatory mediator regulation of TRP channels	1	0.284223
Bile secretion	2	0.233819	Glucagon signaling pathway	1	0.21975
Mineral absorption	2	0.030239	Regulation of lipolysis in adipocytes	1	0.124909
Choline metabolism in cancer	2	0.004561	Renin secretion	1	0.149623
Citrate cycle (TCA cycle)	1	0.173656	Aldosterone synthesis and secretion	1	0.189308
Pentose and glucuronate interconversions	1	0.426284	Alcoholic liver disease	1	0.099495
Fructose and mannose metabolism	1	0.403728	Proximal tubule bicarbonate reclamation	1	0.149623
Fatty acid degradation	1	0.380309	Vitamin digestion and absorption	1	0.311181
Primary bile acid biosynthesis	1	0.362157	Cholesterol metabolism	1	0.090864
Oxidative phosphorylation	1	0.141462	Parkinson disease	1	0.21975
Caffeine metabolism	1	0.189308	Morphine addiction	1	0.073361
Valine, leucine and isoleucine degradation	1	0.330746	Alcoholism	1	0.090864
Valine, leucine and isoleucine biosynthesis	1	0.197025	Kaposi sarcoma-associated herpesvirus infection	1	0.046488
Lysine degradation	1	0.380309	Chemical carcinogenesis - reactive oxygen species	1	0.420724
Phenylalanine metabolism	1	0.374315	Diabetic cardiomyopathy	1	0.311181
Phenylalanine, tyrosine and tryptophan biosynthesis	1	0.277324			

p-value, obtained from the t-test of the substance in this group comparison; Number, the number of differential metabolites in this pathway.

Table S3. Analysis of metabolomic pathways in the control group and sodium salicylate group.

Pathway	Total	Hits	Raw p	Impact	Hits ID
Nitrogen metabolism	9	2	0.033226	0	L-Glutamine, L-Histidine
Histidine metabolism	14	2	0.075649	0.26619	L-Histidine, L-Aspartic acid
Glyoxylate and dicarboxylate metabolism	16	2	0.095705	0.33334	Glycolic acid, Citric acid
Phenylalanine, tyrosine and tryptophan biosynthesis	4	1	0.12599	0.5	L-Phenylalanine
D-Glutamine and D-glutamate metabolism	5	1	0.15498	0	L-Glutamine
Aminoacyl-tRNA biosynthesis	64	4	0.15741	0	L-Histidine, L-Phenylalanine, L-Glutamine, L-Aspartic acid
Alanine, aspartate and glutamate metabolism	23	2	0.17481	0.27667	L-Aspartic acid, L-Glutamine
Taurine and hypotaurine metabolism	7	1	0.21016	0	Taurocholic acid
Ascorbate and aldarate metabolism	9	1	0.26181	0	L-Gulonolactone
Phenylalanine metabolism	9	1	0.26181	0.40741	L-Phenylalanine
Caffeine metabolism	12	1	0.33314	0	7-Methylxanthine
Nicotinate and nicotinamide metabolism	13	1	0.35539	0.2439	NAD
Purine metabolism	68	3	0.39287	0.0118	L-Glutamine, Adenosine, Hypoxanthine
Arginine and proline metabolism	44	2	0.43196	0	L-Glutamine, L-Aspartic acid
beta-Alanine metabolism	17	1	0.43733	0	L-Aspartic acid
Fructose and mannose metabolism	19	1	0.47439	0.06818	Sorbitol
Citrate cycle (TCA cycle)	20	1	0.49201	0.05356	Citric acid
Sphingolipid metabolism	21	1	0.50905	0.14286	Sphinganine
Galactose metabolism	26	1	0.58623	0	Sorbitol
Glutathione metabolism	26	1	0.58623	0.01431	Pyroglutamic acid
Cysteine and methionine metabolism	28	1	0.61366	0.07992	2-Oxo-4-methylthiobutanoic acid
Amino sugar and nucleotide sugar metabolism	37	1	0.71661	0	Glucosamine
Valine, leucine and isoleucine degradation	38	1	0.72624	0	Methylmalonic acid
Biosynthesis of unsaturated fatty acids	42	1	0.76165	0	Icosadienoic acid

Primary bile acid biosynthesis	46	1	0.79257	0.02976	Taurocholic acid

Total, the total number of compounds in the pathway; Hits, the actually matched number from the user uploaded data; Raw p, the original *p*-value calculated from the enrichment analysis; Impact, the pathway impact value calculated from pathway topology analysis; Hits ID, names of differential metabolites.

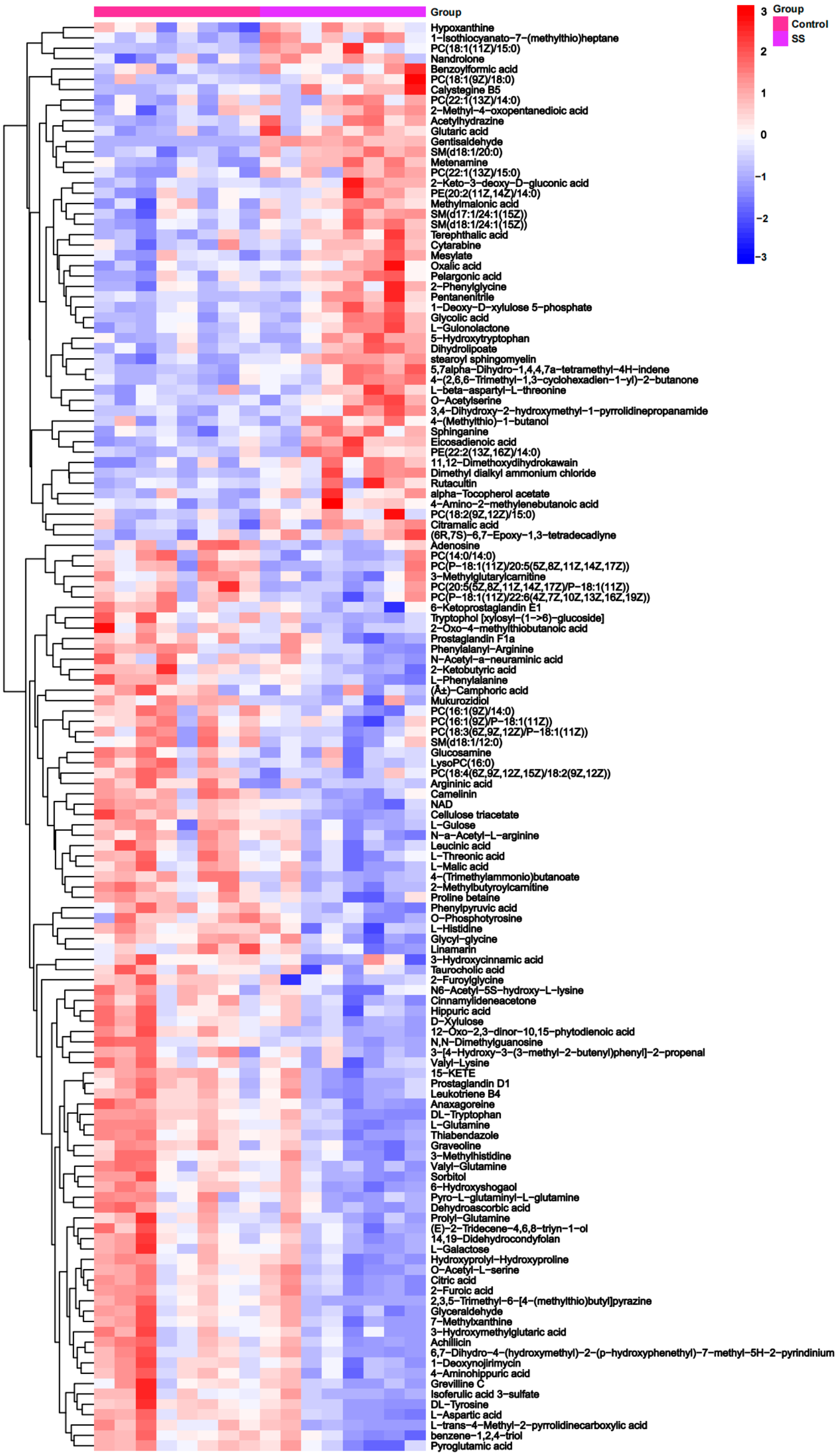

Figure S1. Hierarchical cluster analysis thermogram of differential metabolites in sodium salicylate (SS) sheep sperm of the control group.
